# Using the Chinese herb *Scutellaria barbata* against extensively drug-resistant *Acinetobacter baumannii* infections: in vitro and in vivo studies

**DOI:** 10.1186/s12906-018-2151-7

**Published:** 2018-03-20

**Authors:** Chin-Chuan Tsai, Chi-Shiuan Lin, Chun-Ru Hsu, Chiu-Ming Chang, I-Wei Chang, Li-Wei Lin, Chih-Hsin Hung, Jiun-Ling Wang

**Affiliations:** 1 School of Chinese Medicine for Post-Baccalaureates, Chinese Medicine Department, I-Shou University and E-DA Hospital, Kaoshiung, Taiwan; 2 Department of Medical Research, E-DA Hospital and School of Medicine, I-Shou University, Kaoshiung, Taiwan; 3 Department of Pathology, Taipei Medical University Hospital and , School of Medicine, College of Medicine, Taipei Medical University , Taipei, Taiwan; 4 Department of Chemical Engineering, and Institute of Biotechnology, I-Shou University, Kaoshiung, Taiwan; 5 Department of Internal Medicine, National Cheng Kung University Hospital and College of Medicine,National Cheng Kung University, No. 138, Sheng Li Road, Tainan, 70403 Taiwan

**Keywords:** Multidrug-resistant *Acinetobacter baumannii*, *Scutellaria barbata*, Disc diffusion method, Time-kill curve, Animal model

## Abstract

**Background:**

No animal model studies have been conducted in which the efficacy of herbal compounds has been tested against multidrug-resistant *Acinetobacter baumannii* infections. Very few antibiotics are available for the treatment of pulmonary infections caused by extensively drug-resistant *Acinetobacter baumannii* (XDRAB). To find alternative treatments, traditional Chinese herbs were screened for their antimicrobial potential.

**Methods:**

The present study screened 30 herbs that are traditionally used in Taiwan and that are commonly prescribed for heat clearing and detoxification. The herbs with antibacterial activities were analysed by disc diffusion assays, time-kill assays and a murine lung infection model.

**Results:**

Of the 30 herbs tested, only *Scutellaria barbata* demonstrated 100% in vitro activity against XDRAB. Furthermore, we compared the antibacterial effect of the *S. barbata* extract with that of colistin, and the *S. barbata* extract showed better antibacterial effect. In the XDRAB pneumonia murine model, we compared the antimicrobial effects of the orally administered *S. barbata* extract (200 mg/kg, every 24 h), the intratracheally administered colistin (75,000 U/kg, every 12 h), and the control group. The bacterial load in the lungs of the treatment group that received the oral *S. barbata* extract showed a significant decrease in comparison to that in the lungs of the control group. In addition, histopathological examinations also revealed better resolution of perivascular, peribronchial, and alveolar inflammation in the oral *S. barbata* extract-treated group.

**Conclusions:**

Our in vitro and in vivo data from the animal model support the use of *S. barbata* as an alternate drug to treat XDRAB pulmonary infections. However, detailed animal studies and clinical trials are necessary to establish the clinical utility of *S. barbata* in treating XDRAB pulmonary infections.

## Background

Since 2000, multidrug-resistant *Acinetobacter baumannii* (MDRAB) strains have rapidly emerged, and their prevalence has increased worldwide, including in Taiwan. Currently, MDRAB is one of the most important pathogens associated with nosocomial pneumonia in hospitals, and it can lead to further complications, such as bacteraemia and sepsis. There are a limited number of effective antibiotics to treat MDRAB infections, including colistin and tigecycline [1–3]. However, the efficacy of colistin is limited by its nephrotoxicity and by the development of colistin-resistant MDRAB strains [4, 5]. Moreover, several retrospective studies on the effectiveness of tigecycline against MDRAB infections have suggested that the clinical efficacy of tigecycline-based therapy is still controversial. The development of breakthrough bacteraemia and the emergence of drug resistance during the course of therapy limit the efficacy of tigecycline therapy for MDRAB when used as the single therapeutic agent [3, 6].

A study conducted by Savoia on the potential antimicrobial activity of plant-derived substances suggested that naturally bioactive plant compounds can be a source of new drugs in the future [7]. Some of the active compounds extracted from herbs have shown potential activity against *A. baumannii* and other gram-negative bacteria. Many plant-based natural compounds that show considerable antimicrobial activity against *Escherichia coli* or *Pseudomonas aeruginosa* have not been tested against *A. baumannii* [8]*.* Several medicinal plants extracts such as those from *Calotropis procera*, *Aegle marmelos*, *Actinidia deliciosa* and *Punica granatum* peel or nanomaterial-based therapies have been found to have antimicrobial activity against MDRAB [9–12]. After screening sixty herbal extracts, Miyasaki et al. reported that approximately 30% of the screened herbs displayed potential in vitro antimicrobial activity against MDRAB. The six most active compounds identified from the herbal extracts were ellagic acid from *Rosa rugosa*, norwogonin from *Scutellaria baicalensis,* and chebulagic acid, chebulinic acid, corilagin, and terchebulin from *Terminalia chebula* [13, 14]. However, further attempts to develop potent antimicrobials from plants were not successfully undertaken by pharmaceutical or biotechnology firms. One reason is that antibacterial compounds act more effectively in combination but show much lower efficacy when used in their isolated and purified forms [8].

In this context, searching for effective natural antimicrobial agents from Chinese herbs that have been used for centuries seems to be an alternative solution. Several heat-clearing and detoxifying Chinese herbs have been reported to have anti-inflammatory and antimicrobial effects through different mechanisms of action and on multiple targets [15]. In this study, we screened the commonly used heat-clearing Chinese herbs for activity against MDRAB by in vitro methods. Due to difficulty in conducting randomized controlled clinical trials for MDRAB infection, animal models (using the compounds that showed in vitro efficacy) are usually employed to evaluate the efficacy of test compounds in the treatment of MDRAB infection. There are several reports available in which animal models of *A. baumannii* pneumonia have been used to assess the efficacy of inhaled colistin against MDRAB pneumonia [16, 17], but no animal model studies have been conducted in which the efficacy of herbal compounds against MDRAB infection has been tested. In this study, we evaluated the antimicrobial effect of Chinese herbs against MDRAB infection in a mouse model.

## Methods

### In vitro studies

#### Microorganisms

Thirty-four clinical strains of *A. baumannii* were collected from five medical centres in Taiwan. All the isolates were subjected to MIC testing for various drugs using the CLSI (Clinical and Laboratory Standards Institute) guidelines [18]. Extensively drug-resistant *A. baumannii* (XDRAB) strains were defined as the bacterial isolates resistant to all authorized antibiotics except for tigecycline and polymyxin. There were seventeen XDRAB strains among the isolates.

Genomic DNA was extracted using a standard protocol. The genomic DNA of all 34 strains was digested with *Apa*I and separated by PFGE. Different DNA patterns were seen after PFGE, which indicated that all 34 strains were different genotypes (data not shown).

### Screening of antimicrobial activity

To evaluate the antimicrobial potential of Chinese herbs against XDRAB, we obtained 30 Chinese herbs from a traditional herb store in Kaohsiung City in Taiwan. These herbs are the most commonly prescribed Chinese herbs for heat clearing and detoxification in the Taiwan National Health Insurance Database (Table 1). All herbs included in this study are considered important species for heat clearing and detoxification in traditional Chinese medicine and classical prescription in Taiwan. They have traditionally been used for respiratory-, digestive- and urinary tract infection-related ailments, such as cough and diarrhoea. Modern pharmacological reports have also demonstrated their antimicrobial, anti-inflammatory and analgesic effects, as well as their antitumour effects. Water extracts of these Chinese herbs were prepared using the following procedure: (a) 100 g of the raw herb was ground to a fine powder, (b) the powder was mixed with 1000 mL of distilled water, (c) the water mixture was boiled for 60 min, and (d) the extract was decanted and concentrated in a vacuum evaporator and then frozen to dry before use.

**Table 1:** The 30 traditional Chinese herbal extracts (128 g/L) and the zone of inhibition (mm)

Isolates	Ab019	Ab14	Ab15	Ab16	Ab19	Ab23	Ab26	Ab29	Ab35	Ab39	Ab40	Ab54	TVG55	KM5	Ab21	TVG68	Ab002	Ab010	Ab011	Ab015	Ab021	KM16	KM18	TSG2	TSG4	TSG5	TSG6	TVG52	TVG57	TVG58	TZ1
*Scutellaria baicalensis* (Huang Qin)	–	11	–	12	–	–	–	–	–	–	–	–	–	–	–	–	11	–	–	–	–	–	–	–	–	–	–	–	–	–	–
*Houttuynia cordata* (Yu Xing Cao)	–	–	–	–	–	–	–	–	–	–	–	–	–	–	–	–	–	–	–	–	–	–	–	–	–	–	–	–	–	–	–
*Taraxacum mongolicum* (Pu Gong Ying)	–	–	–	10	–	–	–	–	–	–	–	–	–	–	–	–	–	–	–	–	–	–	–	–	–	–	–	–	–	–	–
*Scrophularia ningpoensis* (Xuan Shen)	–	11	–	–	–	–	–	–	–	–	–	–	–	–	–	–	–	–	–	–	–	–	–	–	–	–	–	–	–	–	–
*Anemarrhena asphodeloides* (Zhi Mu)	–	12	–	10	–	–	–	–	–	–	–	–	–	–	–	–	–	–	–	–	–	–	–	–	–	–	–	–	–	–	–
*Forsythia suspensa* (Lian Qiao)	–	12	–	12	–	–	–	–	–	–	–	–	–	–	–	–	–	–	–	–	–	–	–	–	–	–	–	–	–	–	–
*Rehmannia glutinosa* (Sheng Di Huang)	–	12	–	–	–	–	–	–	–	–	–	–	–	–	–	–	–	–	–	–	–	–	–	–	–	–	–	–	–	–	–
*Belamcanda chinensis* (She Gan)	–	14	–	–	14	–	–	–	–	–	–	–	–	–	–	–	–	–	–	–	–	–	–	–	–	–	–	–	–	–	–
*Paeonia suffruticosa* (Mu Dan Pi)	–	14	–	–	–	–	–	–	–	–	–	–	–	–	–	–	–	–	–	–	–	–	–	–	–	–	–	–	–	–	–
*Phellodendron amurense* (Huang Bo)	–	–	–	–	–	–	–	–	–	–	–	–	–	–	–	–	–	–	–	–	–	–	–	–	–	–	–	–	–	–	–
*Coptis chinensis* (Huang Lian)	–	–	–	–	–	–	–	–	–	–	–	–	–	–	–	–	11	–	–	–	–	–	–	–	–	–	–	–	–	–	–
*Gardenia jasminoides* (Zhi Zi)	–	–	–	–	–	–	–	–	–	–	–	–	–	–	–	–	–	–	–	–	–	–	–	–	–	–	–	–	–	–	–
*Isatis indigotica* (Ban lan Gen)	–	–	–	–	–	–	–	–	–	–	–	–	–	–	–	–	–	–	–	–	–	–	–	–	–	–	–	–	–	–	–
*Smilax glabra* (Tu Fu Ling)	–	–	–	–	–	–	–	–	–	–	–	–	–	–	–	–	–	–	–	–	–	–	–	–	–	–	–	–	–	–	–
*Gypsum fibrosum* (Shi Gao)	–	–	–	–	–	–	–	–	–	–	–	–	–	–	–	–	–	–	–	–	–	–	–	–	–	–	–	–	–	–	–
*Prunella vulgaris* (Xia Ku Cao)	–	–	–	–	–	–	–	–	–	–	–	–	–	–	–	–	–	–	–	–	–	–	–	–	–	–	–	–	–	–	–
*Cassia obtusifolia* (Jue Ming Zi)	–	–	–	–	–	–	–	–	–	–	–	–	–	–	–	–	–	–	–	–	–	–	–	–	–	–	–	–	–	–	–
*Dictamnus dasycarpus* (Bai Xian Pi)	–	–	–	–	–	–	–	–	–	–	–	–	–	–	–	–	–	–	–	–	–	–	–	–	–	–	–	–	–	–	–
*Lycium chinense* (Di Gu Pi)	–	–	–	–	–	–	–	–	–	–	–	–	–	–	–	–	–	–	–	–	11	–	–	–	–	–	–	–	–	–	–
*Torenia concolor* (Dao Di Wu Gong)	–	–	–	–	–	–	–	–	–	–	–	–	–	–	–	–	–	–	–	–	–	–	–	–	–	–	–	–	–	–	–
*Lonicera japonica* (Jin Yin Hua)	–	–	–	–	–	–	–	–	–	–	–	–	–	–	–	–	–	–	–	–	–	–	–	–	–	–	–	–	–	–	–
*Hedyotis diffusa* (Bai Huan She She Cao)	–	–	–	–	–	–	–	–	–	–	–	–	–	–	–	–	–	–	–	–	–	–	–	–	–	–	–	–	–	–	–
*Sophora tokiensis* (Shan Dou Gen)	–	–	–	–	–	–	–	–	–	–	–	–	–	–	–	–	–	–	–	–	–	–	–	–	–	–	–	–	–	–	–
*Isatis indigotica* (Da Qing Ye)	–	–	–	–	–	–	–	–	–	–	–	–	–	–	–	–	–	–	–	–	–	–	–	–	–	–	–	–	–	–	–
*Polygonum cuspidatum* (Hu Zhang)	–	–	–	–	–	–	–	–	–	–	–	–	–	–	–	–	–	–	–	–	–	–	–	–	–	–	–	–	–	–	–
*Patrinia scabiosaefolia* (Bai Jiang Cao)	–	–	–	–	–	–	–	–	–	–	–	–	–	–	–	–	–	–	–	–	–	–	–	–	–	–	–	–	–	–	–
*Haliotis diversicolor* (Shi Jue Ming)	–	–	–	–	–	–	–	–	–	–	–	–	–	–	–	–	–	–	–	–	–	–	–	–	–	–	–	–	–	–	–
*Gentiana scabra* (Long Dan)	–	–	–	–	–	–	–	–	–	–	–	–	–	–	–	–	–	–	–	–	–	–	–	–	–	–	–	–	–	–	–
*Sophora flavescens* (Ku Shen)	–	–	–	–	–	–	–	–	–	–	–	–	–	–	–	–	–	–	–	–	–	–	–	–	–	–	–	–	–	–	–
*Scutellaria barbata* (Ban Zhi Lian)	14	15	18	16	17	17	16	14	18	14	15	16	18	14	17	15	16	16	17	14	16	18	16	18	15	17	17	16	17	16	15

We screened 30 clinical isolates of *A. baumannii* for antimicrobial activity by the disc diffusion method on Muller-Hinton agar plates [19]. Each disc contained 20 μL of the herb extract (128 g/L) that was placed on MHA agar inoculated with 5 × 10^5^ cfu/mL of XDRAB. The zone of inhibition was determined after incubation at 37 °C for 16–18 h.

### Determination of MICs and MBCs

The broth microdilution method was used to determine the MIC (minimum inhibitory concentration) and MBC (minimal bactericidal concentration) [18]. An inoculum containing 5 × 10^5^ cfu/mL of XDRAB in the exponential growth phase was used. The microtiter plates were inoculated with the bacterial suspension and the diluted antimicrobials. After incubating the plates at 37 °C for 16–18 h, the growth of the organism in each well was visually detected. The MIC was defined as the lowest concentration of the antimicrobial agent that completely inhibited the growth of the organism in the microdilution wells as detected by unaided eyes. The MBC was defined as the lowest concentration of the antibacterial agent that resulted in ≥99.9% reduction of the initial bacterial inoculum [18] (Table 2).

**Table 2:** MIC and MBC values of colistin and *S. barbata* extract against various *A. baumannii* strains

Strain	Colistin		*S. barbata* extract	
	MIC/MBC (μg/mL)		MIC/MBC (mg/mL)	
Ab019	4	8	6.4	6.4
Ab14	8	8	6.4	6.4
Ab15	4	4	6.4	6.4
Ab16	8	16	6.4	6.4
Ab19	4	4	6.4	6.4
Ab23	4	8	6.4	12.8
Ab26	4	8	6.4	6.4
Ab29	4	4	6.4	6.4
Ab35	8	16	6.4	6.4
Ab39	8	16	6.4	6.4
Ab40	8	8	6.4	6.4
Ab54	4	4	6.4	6.4
TVG55	4	8	6.4	12.8
KM5	4	8	6.4	6.4
Ab21	8	8	6.4	6.4
TVG68	4	4	6.4	6.4
Ab002	8	8	6.4	6.4
Ab010	4	4	6.4	6.4
Ab011	4	4	6.4	6.4
Ab015	4	4	12.8	12.8
Ab021	4	16	12.8	12.8
KM16	4	8	6.4	12.8
KM18	4	8	6.4	6.4
TSG2	8	8	6.4	6.4
TSG4	4	4	6.4	6.4
TSG5	8	16	6.4	6.4
TSG6	8	16	6.4	6.4
TVG52	4	8	6.4	6.4
TVG57	2	2	6.4	6.4
TVG58	4	8	6.4	6.4
TZ1	16	16	12.8	12.8

### Time-kill curve

Initial inoculums of 5 × 10^5^ cfu/mL XDRAB isolates were prepared. Two sets containing the herb extract of the Chinese herb and colistin at concentrations equal to (1×), twice (2×) and four (4×) times the MIC were employed for each strain. Bacterial growth was measured after 0, 1, 2, 4, 8 and 24 h of incubation by plating on BHI agar and incubating at 37 °C for 16–18 h.

### Plant identification and authentication method

Since only the *Scutellaria barbata* extract showed 100% antimicrobial activity in the in vitro experiments, we sent the herbal extract for authentication by a non-profit organization, the Brion Research Institute of Taiwan. Authentication of Scutellaria barbata was performed according to Doc No. BR3-TE01 Authentication SOP by Non-profit organization Brion Research Institute of Taiwan, New Taipei City, Taiwan. Paraffin method was modified from Taiwan Herbal Pharmacopeia [20]. In brief, raw materials were cutting into smashed powder (about 1 cm). After dehydration with mixture of t-Butanol and alcohol (TBA-series), t-Butanol is generally replaced with a pure wax (called infiltration of paraffin) and then the sample was embedded with paraffin. Sectioning with selected thickness, deparaffinization and staining were carried out and sample was observed under an inverted microscope. Identification of tissue mark meets description of Herba of Scutellaria barbata D. Don of Labiatae family [21].

The authentication of *Scutellaria barbata* was performed according to Doc No. BR3-TE01 Authentication Standard Operation Procedure (SOP). We used the paraffin method for the identification of the leaf. The smashed powder of the raw materials was processed with chloral hydrate or water and observed under an inverted microscope.

### Animal model

#### Mice

According to reference material and our experience, 8–12-week-old mice were ideal for this experiment. Younger mice would be too fragile to withstand such a challenge. Inbred female BALB/c mice, 8 to 12 weeks of age, were purchased from the National Experimental Animal Center (Taipei, Taiwan). The mice were kept in animal cages with a 12 h light/dark cycle, with ad libitum access to food and water. All animal experiments were approved by the Animal Use Protocol IACUC of I-Shou University (IACUC-ISU-102032). Tribromoethanol, an injectable anaesthetic agent used in mice, was used in our animal model. Tribromoethanol is prepared by the mixing 2,2,2-tribromoethyl alcohol with tert-amyl alcohol (SIGMA). The material is mixed by swirling prior to administration and given by IP injection at a dose of 250 mg/Kg. This amounts to 0.5 ml of the above solution to a 25 g mouse. Tribromoethanol is effective and simple to use; it provides rapid induction and deep surgical anaesthesia in mice followed by faster postoperative recovery and low morbidity and mortality [22, 23].

In the end time point of animal experiments, all animals were sacrificed by cervical dislocation with anaesthesia. Post induction of anaesthesia (tribromoethanol, 250 mg/Kg, IP injection), the thumb and index finger are placed on either side of the neck at the base of the skull or, alternatively, a rod is pressed at the base of the skull with the animal lying on a table surface. With the other hand, the bases of the tail or hind limbs are firmly and steadily pulled to cause separation of the cervical vertebrae and spinal cord from the skull.

#### Experimental design

Since only the *Scutellaria barbata* extract showed 100% antimicrobial activity in the in vitro experiments, it was used in the animal studies. The mouse model of *A. baumannii*-associated pneumonia was generated as described previously, with minor modifications [24]. Briefly, anaesthetized mice were suspended vertically and given an intratracheal (i.t.) inoculation of aliquots containing the bacterial suspension (the inoculum dose was 1 × 10^9^ cfu in 50 μL of phosphate-buffered saline (PBS)). After an incubation period of 4 h, all the infected mice were randomly divided into three groups as follows: (1) the control group (without treatment); (2) the colistin treatment group (i.t. administration of colistin at 75000 U/kg every 12 h); and (3) the Sb treatment group (oral administration of *S. barbata* extract at 200 mg/kg every 24 h) [25]. The dose of Sb that we used in this study (200 mg/kg) is the same as the dose used for cancer treatment.

### Pulmonary bacterial loads and histopathological studies

Mice were sacrificed at 72 h post treatment. Homogenized lung samples were examined for pulmonary bacterial loads after 72 h of treatment (*n* = 5 per group). Serial tenfold dilutions of the lung homogenates were prepared in saline, and 0.1 mL of each dilution was spread on LB agar plates. The results were expressed as cfu per homogenized lung. For the histopathological studies, the lungs were fixed immediately in 10% neutral buffered formalin and processed by standard paraffin embedding methods. Sections 4 μm thick were cut, stained with haematoxylin-eosin (HE) or Gram stained, and examined under the light microscope (*n* = 4).

### Statistical analysis

Data are presented as the mean ± standard deviation for each group. Differences in quantitative measurements were assessed by Student’s t-test or one-way or two-way analysis of variance (ANOVA). Differences were considered statistically significant at *P* < 0.05.

## Results

### Screening of antimicrobial activity

**Fig. 1:**
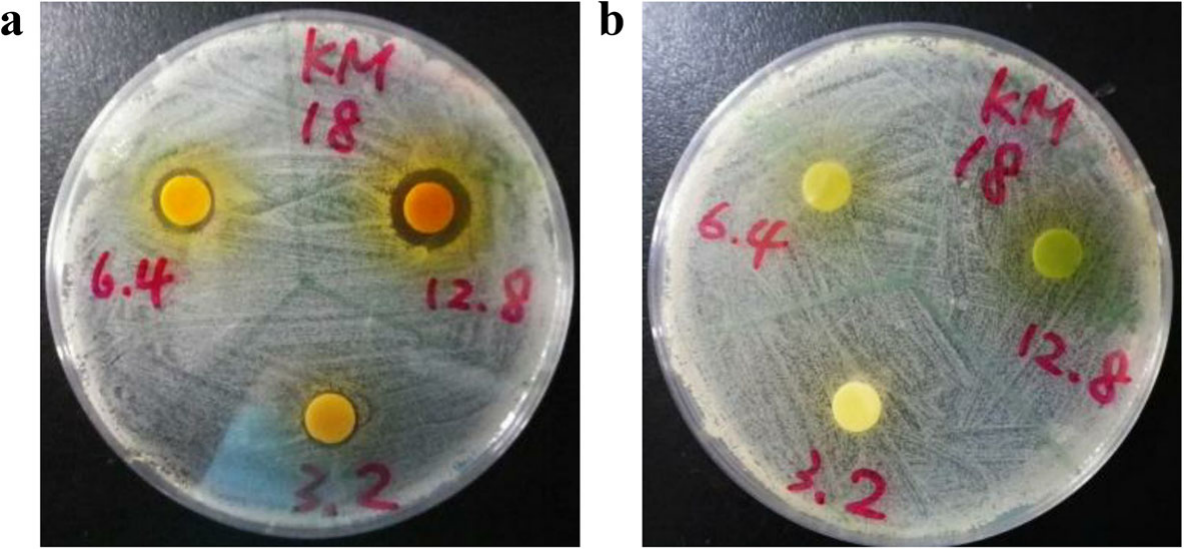
The antibacterial effect of *S. barbata* extract (**a**) and *L. japonica* extract (**b**) assayed at concentrations of 3.2 mg/mL, 6.4 mg/mL and 12.8 mg/mL against *A. baumannii* strain KM18

Among the 30 Chinese herbs screened by the disc diffusion method, only the extract of *Scutellaria barbata* showed 100% antimicrobial activity against the 30 clinical isolates of *A. baumannii*, including the XDRAB strains with different pulsotypes (Table 1 and Fig. 1). The mean diameters of the zones of inhibition ranged from 14 to 18 mm. The MIC and MBC values are shown in Table 1. We also tested five active compounds of *Scutellaria barbata*, including apigenin, baicalin, hispidulin, luteolin, naringenin, wogonin and protocatechuic acid. The data show no antibacterial effect of any single compound at the highest concentration (1280 μg/ml), but we found synergistic effects of several formulas, including hispidulin (640 μg/ml) + protocatechuic acid (640 μg/ml), luteolin (640 μg/ml) + naringenin (640 μg/ml) and pigenin (320 μg/ml) + hispidulin (320 μg/ml) + protocatechuic acid (320 μg/ml) (data not shown).

### Time-kill curve

**Fig. 2:**
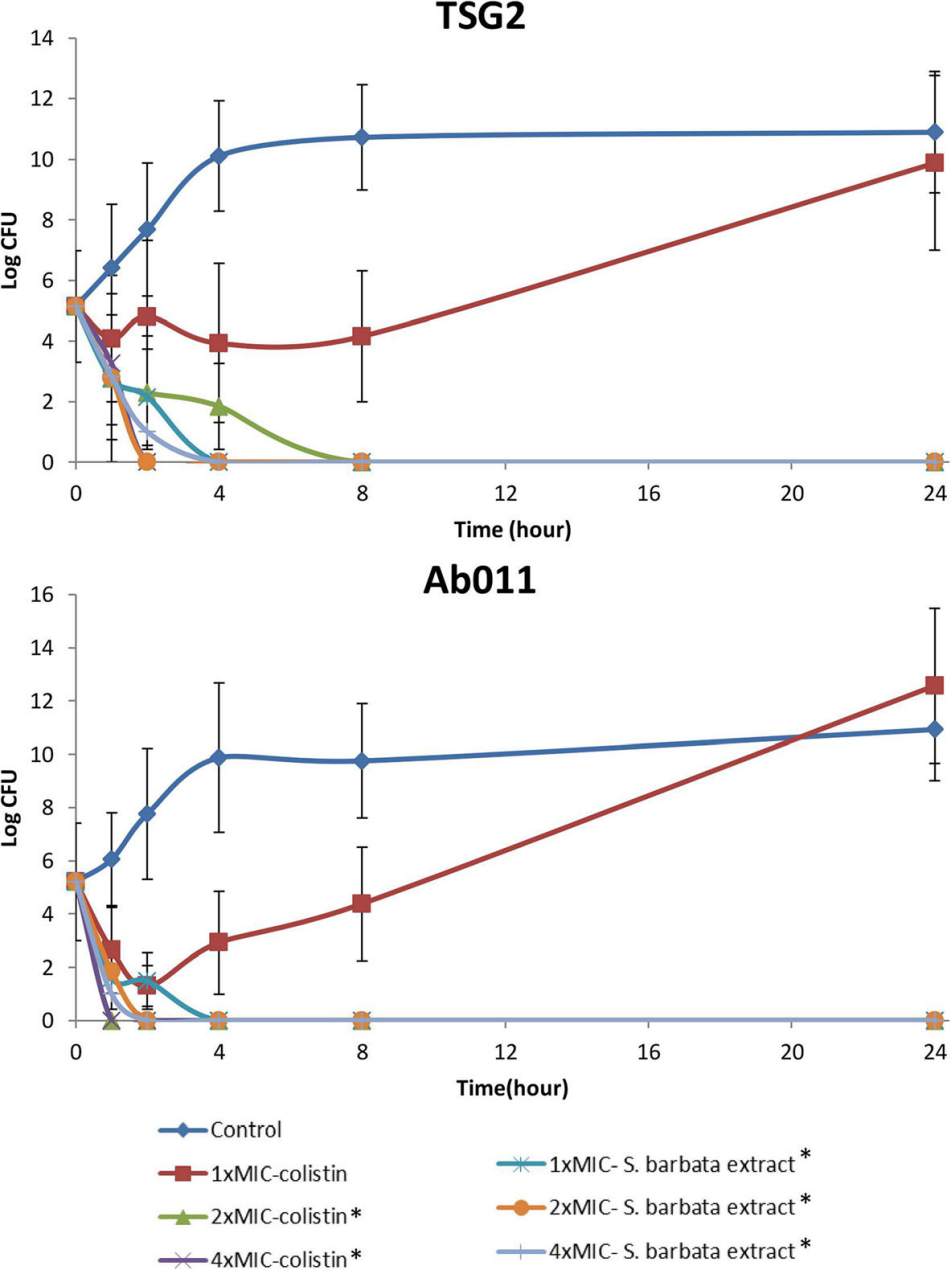
Time-killing curves of colistin and *S. barbata* extract assayed on two selected strains of *A. baumannii* TSG2 and Ab011. “*” Significantly lower bacterial load compared to the control group at 24 h (*P* < 0.05). *n* = 3 per group

The time-kill curve for the *S. barbata* extract showed better bactericidal activity than that of colistin against two selected strains of *A. baumannii,* TSG2 and Ab011. The XDRAB growth was persistent even after 8 h at 1× the MIC concentration of colistin. However, under similar conditions, the *S. barbata* extract could successfully kill the bacteria. Bactericidal activity (≥3 log_10_ cfu/mL) was achieved within 2 to 4 h against two *A. baumannii* isolates treated with the *S. barbata* extract at 1×, 2× and 4× times the MIC concentration (Fig. 2).

### Plant identification

**Fig. 3:**
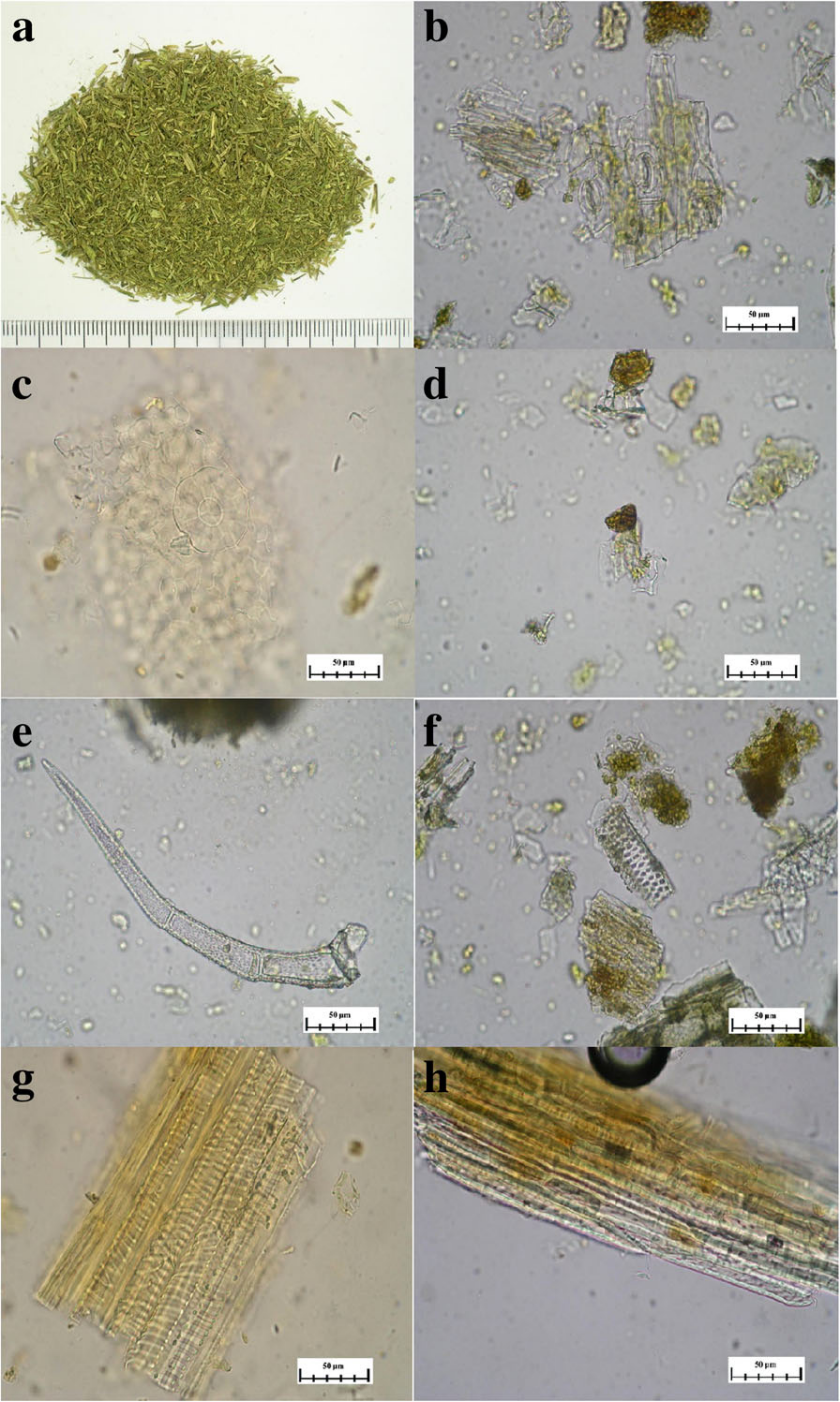
The identification of the *S. barbata* plant

The herbal extract was authenticated as the dried herb of *Scutellaria barbata* D. Don of the Lamiaceae family by the non-profit organization the Brion Research Institute of Taiwan. Briefly, the powder was yellowish-green (Fig. 3-a). The epidermal cells of the leaf walls were slightly curved, and the stoma (Fig. 3-b) were of the diacytic type, with 2–7 subsidiary cells. The glandular scales (Fig. 3-c) were 4- to 8-celled, subrounded or elliptical, and 24–47 μm in diameter. The glandular hairs (Fig. 3-d) consisted of a few-celled head and a single-celled stalk. The non-glandular hairs (Fig. 3-e) consisted of 1–4 cells, with very long apical cells and a fine, warty protuberance on the surface. Bordered-pitted vessels (Fig. 3-f) and spiral vessels (Fig. 3-g) were visible. The fibres (Fig. 3-h) were often in bundles 8–36 μm in diameter that were usually broken, and they had relatively thick walls.

### Histopathological studies of pneumonia

**Fig. 4:**
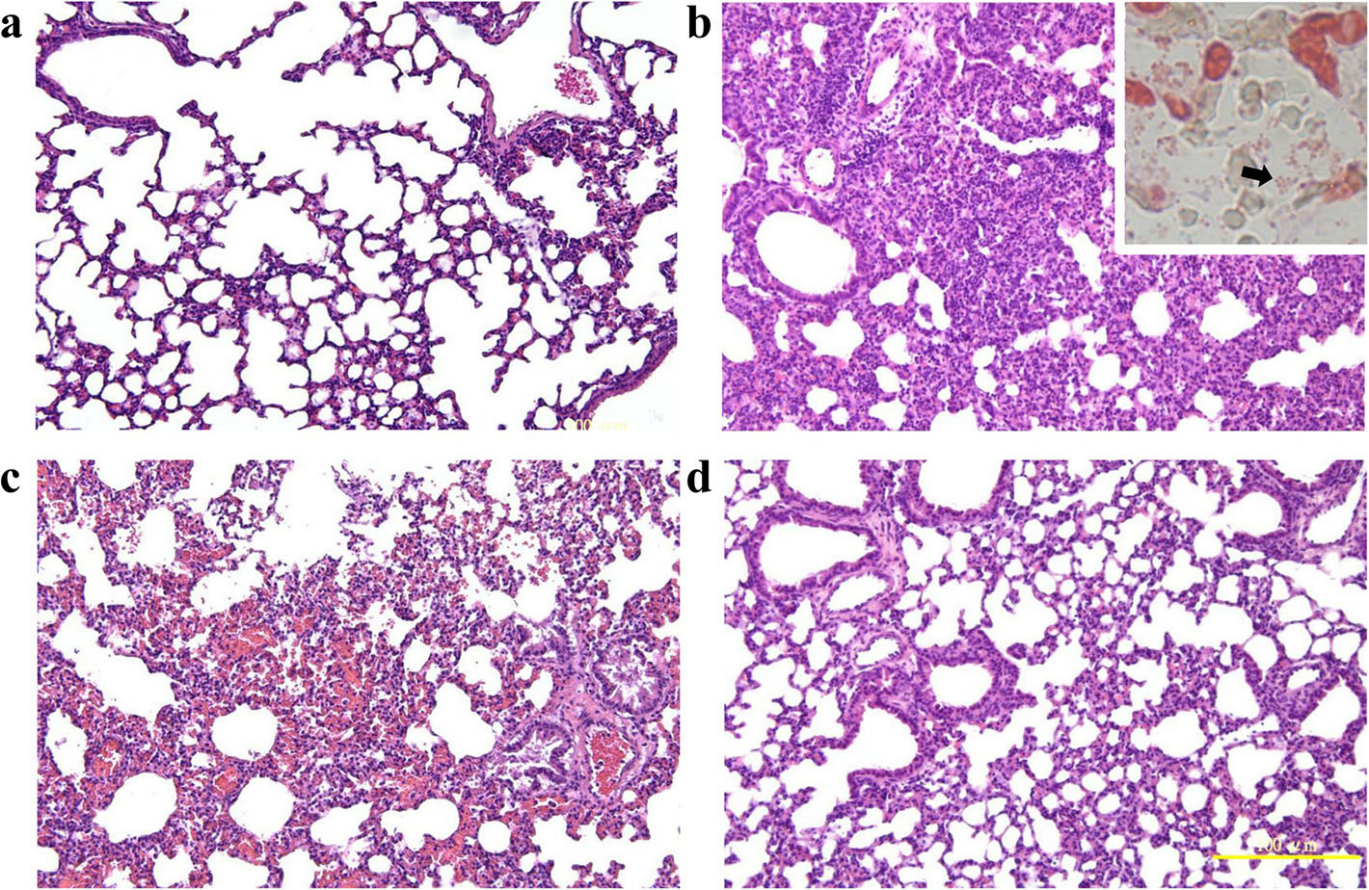
Pathological pulmonary findings after intratracheal inoculation of KM18. Uninfected mice (a, H&E, × 100), control group mice (b, H&E, × 100) and diplococci (arrowhead) in an alveolar sac with Gram staining (right upper panel of b, × 1000), colistin treatment group mice (c, H&E, × 100) and Sb extract treatment group mice (d, H&E, × 100)

Compared with the lungs of uninfected mice (Fig. 4a), the infected lungs revealed acute inflammation, with the infiltration of numerous polymorphonuclear cells and consolidation, which was consistent with pneumonia. The accumulation of invasive gram-negative diplococci was also observed (Fig. 4b). In the colistin treatment group, lung consolidation and infiltration of polymorphonuclear cells were noted (Fig. 4c). However, this inflammatory status abated after Sb extract treatment, indicating the therapeutic efficacy of oral Sb extract against *A. baumannii* infections (Fig. 4d).

### Pulmonary bacterial clearance

**Fig. 5:**
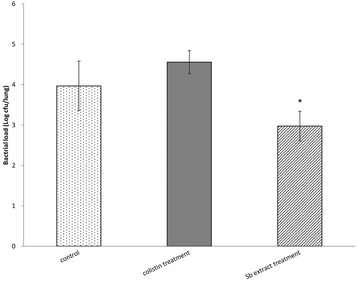
Bacterial load in the lungs of the infected mice without therapy (control group) and infected mice treated with colistin or *S. barbata* (Sb) extract after inoculation with 3.97 ± 0.61, 4.56 ± 0.28 and 2.97 ± 0.36 log CFU/lung (Data are expressed as the mean ± SD of five mice for each group). “*” Significantly lower bacterial load in the *S. barbata* (Sb) extract treatment group compared to the control group (*P* < 0.05)

In the murine model, we compared the efficacy of oral *S. barbata* extract (Sb treatment group) with inhaled colistin (colistin treatment group). All the mice survived until day 3. After sacrificing the mice, the lungs of the mice were examined. It was observed that the bacterial load decreased significantly in the Sb treatment group (*P* < 0.05), whereas a decrease in the bacterial load was not seen in the colistin treatment group or the control group (Fig. 5).

## Discussion

Previous studies have shown that many heat-clearing and detoxifying Chinese herbs have antimicrobial effects [15]. Miyasaki et al. found that plant herbal extracts contain flavones, tannins, and phenolic compounds that are active against MDRAB strains. In their study, they also found that among all the screened herbs, *Scutellaria baicalensis* demonstrated the lowest MIC value against MDRAB [13, 14]. *Scutellaria* species (Lamiaceae) have widely been used in traditional medical systems in China, India, Korea, Japan, European countries, and North America because of their potential antimicrobial activity [26, 27]. In our study, we screened the commonly used heat-clearing and detoxifying Chinese herbs used in Taiwan against MDRAB strains and found that *S. barbata* showed the best antimicrobial activity against MDRAB. The time-kill curve for the *S. barbata* extract against XDRAB showed marked bactericidal activity compared to colistin under similar conditions. Furthermore, in the murine XDRAB pneumonia model, the *S. barbata* extract-treated group showed a decrease in the bacterial load and inflammation in the lungs when compared to the intratracheal colistin-treated group.

*S. barbata* is a perennial herb prevalent in Korea and southern China [28, 29]. Its English common name is barbed skullcap, and its Chinese name is Ban Zhi Lian. *S. barbata* has been used along with other herbs in traditional Chinese and Korean medicine to treat bacterial infections (including carbuncles, cellulitis, and pneumonia), hepatitis, and tumours [30]. Six phenolic compounds, namely, p-coumaric acid, scutellarin, apigenin 5-O-β-glucopyranoside, luteolin, apigenin and 4′-hydroxywogonin, were obtained from *S. barbata* during the phytochemical analyses [31]. Recent studies in many animal models and small clinical trials have demonstrated the antitumour activity of *S. barbata*. The safety and efficacy of an aqueous extract of the aerial parts of *S. barbata* (Bezielle: BZL101) for breast cancer treatment have also been analysed in the Phase I clinical trial undertaken by Bionovo [32–34]. *S. barbata* and *Hedyotis diffusa* are the most commonly prescribed Chinese herbs for breast cancer and post-surgery colon cancer, as reported by Taiwanese nationwide surveys [35, 36]. In addition to anticancer properties, *S. barbata* has been reported to have anti-inflammatory, anticomplementary, antioxidant and antimicrobial properties [36]. A previous study showed that the antimicrobial effect of *S. barbata* is broad spectrum, possessing antibacterial activity against many bacterial strains, including *Escherichia coli*, *Staphylococcus aureus*, *Pseudomonas aeruginosa* and *Salmonella typhimurium*. It was also reported that the antibacterial effect of *S. barbata* is mediated via ROS generation, intracellular protein leakage, and the rupture of bacterial cell membranes [17]. The antimicrobial effect of the essential oil derived from *S. barbata* (50% ethanolic extract) against methicillin-resistant *Staphylococcus aureus* (MRSA) has also been studied. Both of these studies showed that *S. barbata* has better activity against gram-positive cocci than against gram-negative bacteria [37, 38]. Apigenin and luteolin were isolated from the plant as active constituents against MRSA [37]. There are neither in vitro data nor animal model studies available on the utility of *S. barbata* in the treatment of *Acinetobacter* infection, although several animal models of cancer have been reported [39, 40]. A company in China used a combination of lobelia extract (50%) and *S. barbata* extract (50%) and showed that it has 90% efficiency in treating pneumonia caused by mixed a bacterial and viral infection in pigs and in treating mastitis in cows. In addition, they reported that *S. barbata* extract alone has a comparatively lower efficacy (70%) [41]. Another study in China also showed that *S. barbata* can inhibit the expression of the quorum sensing gene involved in *Pseudomonas* infections [42]. In this study, we found that among the commonly used heat-clearing and detoxifying Chinese herbs, *S. barbata* displayed the maximum potency against XDRAB. This finding was further validated by animal model studies. Furthermore, an active component study showed that while no single compound was active against XDRAB, combinations of two or more active compounds displayed considerable antimicrobial activity against XDRAB (data not shown). An explanation for this observation could be that the antibacterially active compounds act effectively in combination but show very low efficacy when used alone [8].

The limitations of this study are as follows: (1) Our in vitro tests showed good activity of *S. barbata* extract against XDRAB; however, the outcome of the tested bacterial isolates cannot conclude that *S. barbata* would also be active against other worldwide clinical isolates. The inclusion of more clinical isolates of *A. baumannii* may improve the generalizability of the study. (2) The animal model study was preliminary, and the ideal dosage needs to be optimized. The dose we used in this study (200 mg/kg) was similar to the dose used in human clinical trials and in the animal model studies of cancer. (3) Inhaled colistin might produce a local effect in the control group. Further animal model studies comparing intravenous and inhaled colistin need to be conducted. (4) In conventional use, the water decoction of the herbs can be administered orally to treat infection or used externally on skin wounds. In this study, we wanted to identify herbs with the potential to treat XDRAB infections in clinical settings. In our future study, we will apply another extraction method (polar and non-polar) to determine the active compounds and antibacterial mechanisms.

## Conclusion

*S. barbata* could be an alternative therapy in the treatment of infection caused by the superbug *Acinetobacter baumannii*. Further human clinical trials are necessary to determine whether it can be used for the treatment of XDRAB pulmonary infection.
